# Modeling the Adaptation of Dairy Cows to Automatic Milking Systems Using Statistical Methods and Machine Learning: Development of the Robotic Adaptability Index

**DOI:** 10.3390/ani16111703

**Published:** 2026-06-02

**Authors:** Dariusz Piwczyński, Wilhelm Grzesiak, Daniel Zaborski, Kamil Siatka

**Affiliations:** 1 Department of Animal Biotechnology and Genetics, Bydgoszcz University of Science and Technology, 85-084 Bydgoszcz, Poland; 2 Department of Biostatistics, Bioinformatics and Animal Research Methods, West Pomeranian University of Technology in Szczecin, 71-270 Szczecin, Poland; wilhelm.grzesiak@zut.edu.pl (W.G.); daniel.zaborski@zut.edu.pl (D.Z.); 3 Department of Animal Breeding and Nutrition, Bydgoszcz University of Science and Technology, 85-084 Bydgoszcz, Poland; kamil.siatka@pbs.edu.pl

**Keywords:** artificial neural networks, adaptation, automatic milking system, booted trees, dairy cows, logistic regression, multiple linear regression, random forest

## Abstract

The objectives of this study were: (1) to identify factors influencing the performance of dairy cows in an automated milking system (AMS); (2) to construct a synthetic robotic adaptability index (RAI) of cows’ adaptation to the AMS; (3) to evaluate the predictive capabilities of traits describing the milking process and RAI; and (4) to compare the predictive power of different modeling approaches. The data on 796 primiparous Polish Holstein–Friesian cows (40,233 milkings) were obtained from the milking robot management system. Milking efficiency (ME), and the average number (AA) and time (AT) of the teat cup attachments and RAI served as predicted variables. Days in milk, and four AMS milking-related and 18 linear conformation traits were used as predictors. The highest predictive ability for ME was achieved with multilayer perceptron (R^2^ = 0.895), followed by linear regression. For AA, AT, and RAI, the highest R^2^ values were obtained for LASSO regression (0.663, 0.642 and 0.670, respectively). The key factors determining milking performance were functional variables, particularly milk flow rate (MilkFlow) and the number of failed milking attempts (Failure), while conformation traits had limited significance. More complex machine learning models do not always lead to improved prediction quality compared to statistical methods, which emphasizes the need for a critical approach to their application in the analysis of production data.

## Simple Summary

Automatic milking systems (AMSs) are widely used on dairy farms, where they influence work organization, cow behavior, and production. However, the development of this technology leads to a significant change in the nature of the interaction between the cow and the production environment, which means that the traditional criteria for assessing the performance value of cows may not fully reflect their functioning under automated milking conditions. Therefore, this study aimed to identify factors influencing the performance of dairy cows in an AMS and to predict selected milking-related traits using different modeling approaches (linear and LASSO regression, tree-based ensemble methods and artificial neural networks). From four predicted variables, the highest predictive performance was obtained for milking efficiency, whereas lower performances were obtained in relation to the number and time of teat cup attachments. The key factors determining milking performance were functional variables, while conformation traits had limited significance. More complex machine learning models do not always lead to improved prediction quality compared to statistical methods, which emphasizes the need for a critical approach to their application in the analysis of production data.

## 1. Introduction

Automatic milking systems (AMSs) are widely used on dairy farms, where they influence work organization, cow behavior, and production monitoring [1]. However, the development of this technology leads to a significant change in the nature of the interaction between the cow and the production environment, which means that the traditional criteria for assessing the performance value of cows may not fully reflect their functioning under automated milking conditions [2]. It should also be noted that the efficiency and functionality of AMSs may differ depending on the technical characteristics of robotic systems, including teat detection accuracy, sensor technologies, robotic arm design, and software algorithms supporting the milking process. These differences may affect both milking performance and the adaptation of cows to AMSs.

The performance of cows in AMSs is determined by complex interactions between physiological, morphological, and behavioral factors [3,4]. Particular importance is attributed to characteristics describing the milking process, such as the milk flow rate, milking duration, and robot utilization efficiency [1,5]. At the same time, a key factor determining the efficiency of the AMS remains the process of attaching the teat cups, the course of which depends on both the animal’s characteristics and its interaction with the technological system [6].

Conformation traits, particularly udder structure, have long been recognized as key factors in assessing the production value of dairy cows, influencing milk yield, udder health, and longevity [7]. In the context of AMSs, however, their significance is more complex, as it also encompasses interaction with the technological system, including the ability to correctly locate teats and the process of automatic attachment of teat cups [8].

Previous research on cow performance in AMSs has focused primarily on the analysis of individual traits, such as milk yield, milking characteristics, or selected behavioral parameters [6,9]. Attempts to integrate this information for a comprehensive assessment of cows’ adaptation to AMSs have been much less frequent, even though this process is multidimensional [10]. As a result, there is still a lack of synthetic indicators integrating different aspects of the milking process, as well as studies comparing the usefulness of statistical and machine learning approaches in the assessment of cows’ adaptation to AMSs [11]. In particular, there is a lack of synthetic indices combining various aspects of the milking process and studies evaluating their utility in predictive modeling [12].

The rapid development of AMSs enables the collection of large sets of production data, which opens up new opportunities for applying advanced data analysis methods, including machine learning techniques [13,14]. These methods allow for the modeling of complex, nonlinear relationships between animal traits and their performance in production systems, as well as the identification of key predictors of the analyzed phenomena. For example, Aerts et al. [5] used decision tree algorithms to model milking efficiency in an AMS, while Dharejo et al. [15] applied machine learning models to predict the occurrence of mastitis based on data from AMSs, achieving high prediction accuracy. Literature reviews also point to the growing importance of modeling and machine learning methods in the analysis of data from AMSs [16].

Despite the growing number of studies utilizing machine learning methods, their advantage over classical statistical methods in the analysis of data from AMSs is not clear cut, particularly regarding model interpretability and the identification of biologically relevant predictors [11,17]. Therefore, there is a need for the systematic evaluation of the usefulness of both analytical approaches, with particular emphasis on their predictive power and the interpretability of results [11,18].

The objectives of this study were four-fold: (1) to identify factors influencing the performance of dairy cows in an AMS, (2) to construct a synthetic index of cows’ adaptation to the AMS (i.e., the robotic adaptability index, RAI) based on principal component analysis (PCA), (3) to evaluate the predictive capabilities of traits describing the milking process and the RAI using statistical methods and machine learning methods, and (4) to compare the predictive power and interpretability of different modeling approaches.

This study employed a comparative approach involving multiple linear regression, least absolute shrinkage and selection operator (LASSO) regression, and selected machine learning algorithms (random forest, extreme gradient boosting—XGBoost—and artificial neural networks), which made it possible to assess the extent to which increasing model complexity translates into improved prediction quality in the analysis of data from AMSs. The objectives related to identifying factors affecting cow performance in AMSs and evaluating the predictive usefulness of different modeling approaches had primarily a scientific and cognitive character. In contrast, the construction of the RAI and the evaluation of predictive models for assessing cow functioning in AMSs had a practical and application-oriented dimension.

## 2. Materials and Methods

### 2.1. Animals and Data

The study was conducted on a dataset comprising 796 primiparous Polish Holstein–Friesian cows maintained on seven commercial dairy farms located in central and eastern Poland (Mazowieckie, Wielkopolskie, Lubelskie, and Podlaskie regions) and equipped with the Lely Astronaut A4 automatic milking system (Lely Industries N.V., Maassluis, The Netherlands). The data were obtained from the milking robot management system and included 40,233 individual milkings. Data were collected over a 12-month period.

The analyzed farms differed in herd size and the number of cows assigned per milking robot, reflecting typical commercial AMS production conditions in Poland. The number of cows assigned per AMS unit ranged from 55 to 58 animals, indicating a comparable workload among the analyzed milking robots. Cows were housed in free-stall barn systems and managed according to standard commercial dairy farming practices. Cows did not have access to pasture or outdoor paddocks during the data collection period. Animals had permanent access to water and were fed a total mixed ration (TMR) balanced according to their nutritional requirements. Resting areas were equipped with bedding materials commonly used in commercial dairy production systems. All farms operated in accordance with national and European Union regulations concerning dairy cattle welfare.

Given the study’s objective, which was to assess the technical adaptation of cows to AMSs at the individual level, data from individual milkings were aggregated to the cow level by calculating average values for specific milking process parameters (Table 1). As a result, each observation in the analysis corresponded to a single cow. This approach also resulted from the fact that conformation traits were recorded once for each cow, whereas AMS-derived variables represented repeated measurements. Without aggregation, the dataset would contain multiple AMS records linked to a single conformation score, leading to pseudo-replication and violation of the independence assumption. Although this approach reduced temporal and within-cow variability, it enabled stable long-term characterization of individual cows’ performance in AMSs.

The dataset included the cow ID, farm code, and a set of traits describing the milking process in the AMS, as well as functional and conformation traits.

The cows’ adaptation to the AMS was assessed based on three variables describing the milking process:

- MilkingEfficiency (ME)—operationally defined as daily milk yield divided by the total box time (time spent by a cow per day in the AMS milking box, [kg/min]).
- AverageNumber (AA)—the average number of attempts to attach the teat cups per milking session [n/milking].
- AverageTime (AT)—the average time required to attach the teat cups during a single milking [s/milking].

Higher ME values indicated a more efficient milking process, while higher AA and AT values indicated a more difficult process of attaching the teat cups.

The analysis also included variables describing the cows’ physiological conditions and the way the milking robot was used (functional characteristics):

- DIM—number of days in milk [day].
- MilkingFreq—average number of milkings per day [n/day].
- MilkFlow—milk flow rate [kg/min].

The analysis also included variables describing cow–robot interaction and behavioral responses in the AMS, including the following:

Refusal—number of refusals to enter the milking robot [n/day].

Failure—number of failed milking attempts [n/day].

Linear conformation scores describing the cows’ body structures and udders were also determined and included in the analysis:

- HeightSacrum—height at the sacrum [cm].
- BodyDepth—body depth [pts].
- ChestWidth—chest width [pts].
- RumpAngle—rump angle [pts].
- RumpWidth—rump width [pts].
- RearLegsSet—rear leg conformation (side view) [pts].
- FootAngle—foot angle [pts].
- RearLegsRearView—rear leg alignment (rear view) [pts].
- ForeAttachment—fore udder attachment [pts].
- RearUdderHeight—rear udder attachment height [pts].
- CentralLigament—central udder ligament [pts].
- UdderDepth—udder depth [pts].
- RearUdderWidth—width of the rear part of the udder [pts].
- RearTeatPlacement—rear teat placement [pts].
- FrontTeatPlacement—front teat placement [pts].
- TeatLength—teat length [pts].
- Angularity—angularity [pts].

These traits were evaluated using a standard linear scale applied in the conformation evaluation of dairy cows. The conformation assessment was carried out by trained experts from the Polish Federation of Cattle Breeders and Dairy Farmers, in accordance with ICAR guidelines.

### 2.2. Development of an Adaptation Index for the AMS

The adaptation of cows to an AMS is a multifactorial process that encompasses both milking efficiency and the course of the teat cup attachment procedure. Therefore, the three variables describing the milking process (ME, AA, and AT) were treated as different aspects of the same biological phenomenon.

PCA was used to integrate the information contained in these variables. The analysis was performed on a correlation matrix, which means that all variables were standardized beforehand. Standardization was performed according to the following formula:

(1) $$Z_{x}=\frac{x_{i}-\bar{x}}{SD_{x}}$$

where

- $x_{i}$—the trait value for the *i*-th cow;
- $\bar{x}$—the population mean (*n* = 796);
- $SD_{x}$—the standard deviation of the trait.

The first principal component (PC) was used to construct the RAI, which was defined as a linear combination of standardized variables:

(2) $$RAI=0.3013\cdot Z_{ME}-0.9694\cdot Z_{ATT}-0.9634\cdot Z_{TIME}$$

where

■ *Z_ME_*—standardized ME value;
■ *Z_ATT_*—standardized AA value;
■ *Z_TIME_*—standardized AT value.

Higher index values indicated greater milking efficiency, fewer attempts to attach the teat cups, and shorter attachment time, and, thus, the better adaptation of the cow to the AMS.

To improve breeding interpretability, the index was subsequently rescaled to a range of 0–100 according to the following formula:

(3) $$RAI_{0-100}=100\times \frac{RAI_{i}-RAI_{min}}{RAI_{max}-RAI_{min}}$$

where *RAI_min_* and *RAI_max_* denote the minimum and maximum values of the index in the analyzed population, respectively.

### 2.3. Predictive Modeling

To assess the predictive capabilities of traits describing cow performance in an AMS, an approach based on predictive modeling was employed. The analysis was conducted for three traits describing the milking process, ME, AA, and AT, as well as for the *RAI* constructed based on them.

The functional traits of cows and conformation traits describing the body and udder structure were used as explanatory variables.

To compare the predictive power of various analytical methods, models of increasing complexity were used.

#### 2.3.1. Multiple Linear Regression

Multiple linear regression was applied as a reference model to describe the linear relationships between the analyzed variables. The model took the following form:

(4) $$Y_{i}=\beta_{0}+\beta_{1}X_{1i}+\cdots +\beta_{k}X_{ki}+\varepsilon_{i}$$

where *Y*_i_ denotes the dependent variable under analysis (ME, AA, AT, or *RAI*), *X*_1i_…*X*_ki_ denote the explanatory variables, *β*_0_ is the intercept, *β_i_* is the regression coefficient, and *ε* is the random error.

#### 2.3.2. LASSO Regression

Least absolute shrinkage and selection operator (LASSO) regression was used to select the most important predictors and to mitigate the problem of multicollinearity. The method involves estimating regression coefficients with an additional penalty term (λ∑∣*β*_j_∣), which leads to the reduction in some coefficients to zero (*β*_j_ denotes the regression coefficient for the *j*th explanatory variable, while λ is the regularization parameter controlling the strength of the penalty imposed on the regression coefficient values; the value of the regularization parameter λ was determined automatically based on cross-validation).

#### 2.3.3. Random Forest

Random forest (RF) is an ensemble algorithm based on the construction of multiple decision trees created on random subsamples of the data. The final prediction is the average of the predictions from all the trees included in the model. The number of randomly selected predictors for each split in a single tree was five, and the proportion of randomly selected cases for each tree was 50%. In addition, 30% of the cases from the training set were randomly assigned to the validation set, which was used to determine the optimal number of component trees and prevent overfitting, while the maximum number of component trees was 1000. The minimum size of the parent and child nodes was five, with this parameter controlling the minimum number of cases in a tree node required for its splitting and thus limiting the tree’s complexity (the number of nodes and levels). New component trees were added to the model until the mean squared error on the validation set did not fall below 5.0% over ten consecutive iterations (of adding new component trees to the model) [19].

#### 2.3.4. Extreme Gradient Boosting

Extreme gradient boosting (XGBoost) is a boosting algorithm based on the sequential construction of decision trees that minimize the loss function through gradient optimization. The learning rate, which determines the weight with which component trees were added to the model, was 0.1, while the proportion of randomly selected cases for each component tree was 50.0%. Furthermore, 30.0% of the cases from the training set were randomly assigned to the validation set, and the maximum number of component trees was 1000. The minimum size of the parent and child nodes was five and one, respectively, while the maximum number of nodes in a component tree was three. New trees were added to the model until the lowest mean polynomial error on the validation set was achieved [19]. In both tree-based methods, the relative importance of predictors was determined based on the total reduction in prediction error (e.g., MSE) obtained as a result of splits using a given variable, summed across all nodes and trees, and then expressed relative to the maximum value.

#### 2.3.5. Artificial Neural Networks

The Broyden–Fletcher–Goldfarb–Shanno algorithm was used to train the multilayer perceptron (MLP) [20]. Statistica’s automatic designer enabled the automatic selection of the optimal network structure and parameters (the number of neurons in the hidden layer, type of postsynaptic potential function and activation function, number of training epochs, etc.). All networks were trained until the lowest possible error (sum of squares) was achieved on the validation set (a portion of the entire dataset used to prevent overfitting). The relative importance of predictors was determined based on the error magnitude for the network with the predictor removed. The predictors were then sorted by the value of the error ratios (error for the model with the predictor removed relative to the full model).

For each of the analyzed dependent variables (ME, AA, AT, and RAI), all of the aforementioned models were fitted using the same set of explanatory variables.

To evaluate the predictive ability of the models, 10-fold cross-validation was used. The data were divided into ten subsets, nine of which were used to train the model, and one for testing. The procedure was repeated ten times so that each subset served as the test set exactly once.

The predictive ability of the models was assessed based on the coefficient of determination (R^2^), the root mean square error (RMSE), and the mean absolute error (MAE).

The models were compared in terms of prediction accuracy to determine which approach best described the relationships between the analyzed features.

For the multiple regression models, standardized *β* coefficients are presented, whereas for the LASSO models, unstandardized coefficients are shown, which resulted from the different assumptions and methods of parameter estimation in both approaches.

## 3. Results

### 3.1. Principal Component Analysis (PCA) for the AMS Traits

To integrate the information contained in the three variables describing the milking process in the AMS, a PCA was performed. The analysis included the ME, AA, and AT variables after their prior standardization. The results of the PCA, including eigenvalues and the proportion of explained variance, are presented in Table 2.

The first principal component (PC1) explained 65.3% of the total variance in the analyzed variables (Table 2). The highest factor loadings for PC1 were obtained for AA (0.969) and AT (0.963), while ME was characterized by a significantly lower contribution (−0.301), suggesting that this component primarily described the teat cup attachment process (Table 3). Consequently, the constructed index was influenced mainly by variables related to teat cup attachment.

### 3.2. Robotic Adaptability Index (RAI)

The RAI was constructed based on factor loadings. High index values corresponded to a more efficient milking process and better adaptation of cows to the AMS, as evidenced by the correlation analysis of the RAI with the analyzed variables presented in Table 4. The relationships between the analyzed variables describing the milking process are presented in Table 5.

### 3.3. Prediction Model Results

The predictive accuracy results for all analyzed models are presented in Table 6. The highest predictive ability for milking efficiency (ME) was obtained for the MLP model (R^2^ = 0.895), with very similar values achieved for linear regression, LASSO, and XGBoost.

For variables describing the teat cup attachment process (AA and AT), the highest coefficients of determination were obtained for the LASSO model (0.663 and 0.642, respectively), indicating its superiority in modeling these traits.

For the RAI, the best results were also obtained for the LASSO model (R^2^ = 0.670), while the other models demonstrated similar, moderate predictive ability.

#### 3.3.1. Multiple Regression Model

The multiple regression model for milking efficiency (ME) demonstrated very high explanatory ability (R^2^ = 0.879). For AA and AT, these values were significantly lower (0.198 and 0.180, respectively), while for the RAI they were 0.225 (Table 7).

The strongest predictor of milking efficiency was milk release speed (MilkFlow), while the number of unsuccessful milking attempts (failures) played a dominant role in the AA and AT models.

#### 3.3.2. LASSO Model

Using LASSO regression improved the predictive ability of the models for the AA, AT, and RAI variables compared to linear regression.

The highest R^2^ values were obtained for AA (0.663), AT (0.642), and RAI (0.670), while maintaining similar performance for ME (R^2^ = 0.877).

The results of the LASSO model, including regularization parameters, prediction performance, and key predictors, are presented in Table 8.

The functional variables, particularly MilkFlow and Failure, remained the most important predictors.

#### 3.3.3. Machine Learning Models

The final RF models for ME, AA, AT, and RAI included 1000, 380, 290 and 1000 component trees, respectively. The XGBoost models for ME, AA, AT, and RAI included 302, 138, 138 and 138 component trees, respectively. The best MLP networks for ME, AA, AT, and RAI had the 22-11-1, 22-7-1, 22-10-1, and 22-7-1 architectures, respectively (the number of neurons in the input, hidden, and output layers, respectively). The hidden layer of the MLP network used a logistic (for ME and AT) or exponential (for AA and RAI) activation function, while the output layer used a linear (for ME), logistic (for AA and RAI), or hyperbolic tangent (for AT) function. The Broyden–Fletcher–Goldfarb–Shanno algorithm took 14, 16, 11, and 15 epochs to converge for ME, AA, AT, and RAI, respectively. In general, models based on machine learning methods (RF, XGBoost, and MLP) demonstrated varying predictive ability.

In the case of ME, the highest accuracy was achieved for the MLP model, but the differences between the models were small. For the AA and AT variables, these models did not demonstrate a clear advantage over LASSO, and the obtained R^2^ values were similar to or lower than those of the linear models. For the RAI, all models achieved moderate predictive accuracy, with no clear advantage for nonlinear methods.

### 3.4. Predictor Importance

The relative predictor importance for the RF, XGBoost, and MLP models is presented in Table 9.

In all analyzed models, the most important predictors were those describing cow performance in the AMS. For ME, MilkFlow played a dominant role, while for AA and AT, the number of milking failures (Failures) was the most influential factor. For RAI, both Failure and MilkFlow were crucial, indicating their central role in determining cow adaptation to the AMS.

The study results clearly indicated that the key factors determining milking performance in the AMS are functional variables, particularly milking speed (MilkFlow) and the number of milking failures (Failures).

## 4. Discussion

The aggregation of individual milking data to the cow level enabled the characterization of stable, long-term differences between animals and reduced the influence of short-term variability related to lactation stage or environmental conditions. Such an approach is commonly used in studies focused on the general functioning of cows in AMSs and facilitates the comparison of animals at the individual level [1,3].

At the same time, it should be emphasized that data aggregation leads to the loss of information regarding within-cow variability and does not account for the hierarchical structure of the data (individual milkings nested within cows). Consequently, the obtained results describe the average level of cow performance rather than the temporal dynamics of the milking process [21]. Similar limitations have been highlighted in studies based on AMS data, which indicate the importance of longitudinal and multilevel approaches for a more comprehensive analysis of production processes and cow behavior over time [14,16]. Nevertheless, the choice of this analytical approach was consistent with the primary objective of the present study, which was to evaluate long-term adaptation of cows to AMSs at the individual level rather than to model variability between single milking events. Future studies should therefore incorporate longitudinal or mixed-effects modeling approaches that allow simultaneous consideration of within- and between-cow variability.

One of the main goals of this study was to construct a synthetic index describing cow adaptation to AMSs (RAI). Principal component analysis enabled dimensionality reduction and the integration of information contained in three variables describing the milking process. The results indicate that the RAI was determined primarily by variables related to the teat cup attachment process (AA and AT), whereas the contribution of milking efficiency (ME) was relatively smaller. Therefore, the proposed index should be interpreted mainly as an indicator of technical adaptation to AMSs associated with the efficiency and stability of teat cup attachment rather than as a universal measure of technical adaptation to robotic milking systems.

Attempts have been made in the literature to synthetically assess cow performance in AMSs, including milking efficiency indicators [5], characteristics of the milking process, and the classification of animals according to their performance in robotic milking systems [6]. However, these approaches have mainly focused on the analysis of individual variables or their modeling, whereas integrated indicators describing the multidimensional nature of cow adaptation to AMSs remain relatively rare in the literature. From a practical perspective, this aspect is particularly important because problems related to teat cup attachment are among the major factors limiting the efficiency and functionality of AMSs [5,6].

The obtained results of the predictive models indicate a clear difference in the modeling ability of the analyzed traits. In the case of milking efficiency (ME), all the approaches used, both statistical and machine learning-based, were characterized by high prediction performance. This suggests that the relationships determining this trait are largely linear and can be effectively modeled using classical statistical methods [13,22]. A different situation was observed for the variables describing the teat cup attachment process (AA and AT) and RAI. The very high correlation observed between AA and AT additionally confirms that both variables describe closely related aspects of the teat cup attachment procedure, which justified their integration using PCA.

Prolonged teat cup attachment time may negatively affect milking dynamics and the neurohormonal mechanisms associated with milk ejection, which may consequently reduce the efficiency of the milking process. The observed variability of attachment-related traits may therefore reflect differences in cow morphology, teat placement, udder conformation, and cow–robot interaction during milking.

The highest predictive ability for these traits was obtained for the LASSO model, while more complex machine learning models (RF, XGBoost, MLP) did not demonstrate a clear advantage. These results are consistent with observations presented in the literature, which emphasize that in the case of data with moderate size and high multicollinearity of variables, regularization methods can outperform more complex machine learning algorithms [14].

The lack of a clear advantage of machine learning models over statistical methods may be due to the characteristics of the analyzed dataset. First, the number of observations (*n* = 796) was relatively small in terms of the use of nonlinear models, which require larger datasets for effective training and capturing complex relationships [23]. Second, the explanatory variables exhibited significant multicollinearity, favoring the use of regularization methods such as LASSO regression, which enables the simultaneous selection of predictors and stabilization of parameter estimates [24]. Third, the analysis showed that the relationships between the analyzed variables were largely linear or quasi-linear, limiting the potential advantage of more complex machine learning models. Under such conditions, simpler statistical models can achieve comparable or even higher prediction performance while maintaining greater interpretability [22]. Similar conclusions are presented in reviews on the application of machine learning in agriculture, indicating that the advantage of machine learning models is not universal and strongly depends on the characteristics of the data [13,16].

In addition, the relatively small number of cows and the use of data originating from a limited number of farms may have limited the ability of more complex nonlinear models, such as MLP and XGBoost, to fully exploit their predictive potential. Although the dataset included more than 40,000 individual milking events, aggregation to the cow level reduced the effective number of observations used for model training. Moreover, the absence of an independent external test dataset means that the obtained results should be interpreted primarily as an evaluation of the relative performance of the analyzed models rather than their absolute generalization ability. Future studies should therefore include inter-farm validation using larger and more heterogeneous datasets differing in herd management and AMS configuration.

It should also be noted that only primiparous cows were included in the analysis, which reduced the biological variability associated with parity but may limit the direct applicability of the results to multiparous animals.

An additional limitation of the present study is the lack of information regarding udder health parameters, particularly somatic cell count (SCC), which is an important indicator of milk quality, mastitis risk, and cow functioning in AMSs. The primary objective of the present study was focused on modeling technical and functional aspects of cow adaptation to AMSs; therefore, health-related variables were not included in the predictive analyses. Future studies should integrate production, behavioral, and udder health data to provide a more comprehensive assessment of cow performance in AMSs.

Predictor importance analysis clearly demonstrated that functional variables, particularly milking speed (MilkFlow) and the number of unsuccessful milking attempts (Failures), played a key role in determining milking performance in an AMS. These results are consistent with previous studies indicating that cow behavior and their interaction with the AMS are fundamental to milking efficiency [1,9].

The significance of conformation traits also proved limited, suggesting that functional variables and cow–robot interaction indicators (Refusal, Failure) played a greater role in AMS performance than classical morphological traits.

The relatively limited importance of conformation traits observed in the present study may result from both biological and technological factors. Modern AMSs are increasingly capable of compensating for moderate morphological differences between cows due to improvements in sensor precision, teat localization systems, and robotic arm functionality [3,11]. Consequently, moderate variation in udder and body conformation may currently have a smaller impact on milking performance than in earlier generations of robotic milking systems.

In contrast, functional and behavioral traits associated with cow–robot interaction, such as milk flow characteristics, milking frequency, and unsuccessful milking attempts, may better reflect the actual efficiency of cow adaptation to AMSs under commercial production conditions [1,5]. This may explain why variables directly describing cow performance within the AMS environment showed greater predictive power than classical conformation traits traditionally considered important in dairy cattle evaluation.

These findings are consistent with recent studies demonstrating the growing importance of behavioral and sensor-derived data in the analysis of cow performance within precision livestock farming systems [8,14]. The methods used to assess predictor importance allowed the identification of variables with the greatest predictive power; however, they did not provide a complete interpretation of the direction and nature of relationships between variables. This limitation is typical of more complex machine learning models, which often achieve high predictive performance at the expense of interpretability [25,26]. In breeding applications, where understanding biological mechanisms is crucial, this may limit their practical usefulness.

It should also be noted that the current analysis did not use an independent test set, and the assessment of the models’ predictive ability was based solely on 10-fold cross-validation. Although this method is widely used and allows for effective utilization of available data [23], it may lead to optimistic estimates of predictive performance, particularly in more complex models [22]. Therefore, future studies should include independent external validation datasets to enable a more reliable assessment of model generalization ability [14,16]. Finally, it should be mentioned that only primiparous cows were included in the analysis, which reduced the biological variability associated with parity but may limit the direct applicability of the results to multiparous animals.

## 5. Conclusions

In summary, the obtained results indicate that functional variables and indicators describing cow–robot interaction are crucial in the analysis of AMS data. It should be added that more complex machine learning models do not always lead to improved prediction quality compared to classical statistical methods, which emphasizes the need for a critical and data-oriented approach to their application in the analysis of production data.

From a practical perspective, the obtained results indicate that functional and behavioral traits, particularly milk flow rate and the number of failed milking attempts, may serve as useful indicators for monitoring cow performance in AMSs. These variables may support herd management decisions and could potentially be incorporated into selection strategies aimed at improving cow adaptation to AMSs.

## Figures and Tables

**Table 1:** Characteristics of the analyzed traits.

Variable	Mean	SD	Min	Max
Days in milk (DIM)	93.09	58.24	5	289
ME [kg/min]	1.56	0.39	0.69	2.73
AA [n/milking]	1.40	0.31	1.00	3.08
AT [s/milking]	11.48	6.18	3.00	48.58
Refusal, n/day	1.87	1.63	0	8.16
Failure, n/day	0.08	0.15	0	2.25
MilkingFreq, n/day	2.94	0.62	1.29	5.00
MilkFlow [kg/min]	2.39	0.79	1.03	4.86
HeightSacrum [cm]	145.38	4.10	134	160
BodyDepth [pts]	6.23	1.16	1	9
ChestWidth [pts]	5.20	1.31	1	9
RumpAngle [pts]	5.27	1.11	1	9
RumpWidth [pts]	5.35	1.05	2	9
RearLegsSet [pts]	5.59	1.01	1	9
FootAngle [pts]	5.11	1.32	1	9
RearLegsRearView [pts]	5.63	1.31	1	9
ForeAttachment [pts]	5.50	1.01	1	9
RearUdderHeight [pts]	5.92	0.87	3	9
CentralLigament [pts]	5.85	1.30	1	9
UdderDepth [pts]	6.04	1.21	1	9
RearUdderWidth [pts]	5.25	0.96	2	9
RearTeatPlacement [pts]	6.59	1.13	1	9
FrontTeatPlacement [pts]	5.24	0.98	1	9
TeatLength [pts]	4.71	1.13	1	9
Angularity [pts]	6.21	1.01	2	9

ME—milking efficiency; AA—average number of teat cup attachments; AT—average time of teat cup attachments; and pts—points used in linear conformation evaluation.

**Table 2:** Principal component analysis (PCA) results.

Component	Eigenvalue	Explained Variance (%)	Cumulative Variance (%)
PC1	1.959	65.29	65.3
PC2	0.954	31.81	97.1
PC3	0.087	2.90	100.0

PC—principal component.

**Table 3:** Principal component analysis (PCA) factor loadings.

Variable	PC1	PC2	PC3
ME	−0.301326	0.953480	−0.008861
AA	0.969438	0.127946	−0.209331
AT	0.963359	0.169483	0.207881

ME—milking efficiency; AA—average number of teat cup attachments; AT—average time of teat cup attachments; and PC—principal component.

**Table 4:** Correlations of the robotic adaptability index (RAI) with individual variables.

Variable	RAI	*p*-Value
ME	0.3013	0.000
AA	−0.9694	0.000
AT	−0.9634	0.000
Days in milk	0.1041	0.003
Refusal	0.0439	0.216
Failure	−0.3919	0.00
MilkingFreq	0.1082	0.002
MilkFlow	0.2136	0.000
HeightSacrum	−0.0203	0.568
BodyDepth	0.0002	0.995
ChestWidth	−0.0061	0.865
RumpAngle	−0.1059	0.003
RumpWidth	0.0081	0.819
RearLegsSet	0.0239	0.501
FootAngle	−0.0414	0.243
RearLegsRearView	−0.0539	0.129
ForeAttachment	0.0643	0.070
RearUdderHeight	0.0092	0.795
CentralLigament	0.0110	0.758
UdderDepth	−0.0236	0.506
RearUdderWidth	−0.0212	0.551
RearTeatPlacement	−0.0365	0.304
FrontTeatPlacement	−0.0290	0.413
TeatLength	−0.0157	0.658
Angularity	−0.0273	0.441

ME—milking efficiency; AA—average number of teat cup attachments; and AT—average time of teat cup attachments.

**Table 5:** Correlations between the AMS characteristics (all correlations are statistically significant, *p* < 0.01).

Variable	ME	AA	AT
ME		−0.168	−0.131
AA	−0.168		0.912
AT	−0.131	0.912	

ME—milking efficiency; AA—average number of teat cup attachments; and AT—average time of teat cup attachments.

**Table 6:** Comparison of prediction model performance.

Variable	R^2^	RMSE	MAE
Multiple linear regression			
ME	0.879	0.136	0.098
AA	0.198	0.279	0.196
AT	0.180	5.591	3.979
RAI	0.225	1.728	1.221
LASSO			
ME	0.877	0.137	0.098
AA	0.663	0.280	0.198
AT	0.642	5.632	4.017
RAI	0.670	1.755	1.252
Random forest (RF)			
ME	0.839	0.231	0.175
AA	0.210	0.286	0.206
AT	0.208	5.651	4.141
RAI	0.246	1.762	1.283
Extreme gradient boosting (XGBoost)			
ME	0.871	0.140	0.101
AA	0.160	0.293	0.209
AT	0.154	5.755	4.186
RAI	0.180	1.811	1.307
Multilayer perceptron (MLP)			
ME	0.895	0.126	0.091
AA	0.181	0.285	0.207
AT	0.162	5.702	4.158
RAI	0.228	1.745	1.266

ME—milking efficiency; AA—average number of teat cup attachments; AT—average time of teat cup attachments; RAI—robotic adaptability index; RMSE—root mean square error; and MAE—mean absolute error.

**Table 7:** Multiple linear regression model—performance and significant predictors (standardized *β* coefficients).

Variable	R^2^	RMSE	MAE	Significant Predictors (*β*)
ME	0.879	0.136	0.098	MilkFlow (0.881 ***), Failure (−0.088 ***), Refusal (−0.118 ***), MilkingFreq (−0.041 *), RearUdderWidth (0.077 ***), DIM (−0.033 *), RearTeatPlacement (−0.031 *)
AA	0.198	0.279	0.196	Failure (0.364 ***), MilkingFreq (−0.110 *), DIM (−0.096 **), RearUdderWidth (0.096 *), MilkFlow (−0.084 *), FootAngle (0.071 *)
AT	0.18	5.591	3.979	Failure (0.354 ***), DIM (−0.106 **), RearUdderWidth (0.106 **)
RAI	0.225	1.728	1.221	Failure (−0.368 ***), MilkFlow (0.193 ***), DIM (0.095 **), MilkingFreq (0.088 *), RearUdderWidth (−0.088 *)

* *p* < 0.05; ** *p* < 0.01; and *** *p* < 0.001. ME—milking efficiency; AA—average number of teat cup attachments; AT—average time of teat cup attachments; RAI—robotic adaptability index; RMSE—root mean square error; MAE—mean absolute error; and DIM—days in milk.

**Table 8:** LASSO model—regularization parameters, performance, and regression coefficients.

Variable	λ	Number of Predictors	R^2^	RMSE	MAE	Most Important Predictors (*β*)
ME	0.0028	17	0.877	0.1369	0.0984	MilkFlow (0.437), Failure (−0.231), MilkingFreq (−0.066), RearUdderWidth (0.0319)
AA	0.0116	9	0.6627	0.2801	0.1976	Failure (0.684), MilkingFreq (−0.038), MilkFlow (−0.018), RearUdderWidth (0.0066)
AT	0.1683	9	0.6423	5.6316	4.017	Failure (13.503), MilkingFreq (−0.755), RearUdderWidth (0.300)
RAI	0.0324	14	0.6701	1.7552	1.2515	Failure (−4.578), MilkFlow (0.440), MilkingFreq (0.239), RearUdderWidth (−0.089)

The *β* coefficients are presented in unstandardized form. ME—milking efficiency; AA—average number of teat cup attachments; AT—average time of teat cup attachments; RAI—robotic adaptability index; RMSE—root mean square error; and MAE—mean absolute error.

**Table 9:** Relative predictor importance for XGBoost, RF, and MLP.

Variable	XGBoost				RF				MLP			
	ME	AA	AT	RAI	ME	AA	AT	RAI	ME	AA	AT	RAI
MilkFlow	1 *	7	6	2	1	4	5	2	1	6	22	2
Refusal	2	5	4	7	2	5	6	6	2	12	21	14
Failure	3	1	1	1	3	1	1	1	4	1	1	1
RearUdderWidth	4	13	12	13	7	13	11	12	3	3	3	5
MilkingFreq	5	2	3	3	4	3	3	4	6	2	2	3
HeightSacrum	6	4	2	4	6	6	4	5	11	17	19	22
RearLegsRearView	7	10	10	10	10	12	16	17	9	15	20	7
CentralLigament	8	18	15	17	15	9	20	10	13	7	15	12
DIM	9	3	7	5	5	2	2	3	5	4	4	4
RearTeatPlacement	10	20	16	16	8	20	19	22	8	19	5	8
ChestWidth	11	14	8	11	11	11	13	13	10	16	16	21
RumpWidth	12	12	13	15	19	18	18	18	15	10	11	6
BodyDepth	13	19	18	22	22	21	22	19	19	18	12	18
RearLegsSet	14	11	14	12	18	22	15	21	14	22	18	20
FrontTeatPlacement	15	16	22	18	16	15	21	15	12	13	14	19
FootAngle	16	8	9	8	14	16	14	14	21	9	9	15
Angularity	17	21	20	20	13	14	10	16	18	5	17	11
RearUdderHeight	18	22	17	21	20	19	17	20	16	8	13	17
ForeAttachment	19	15	11	9	17	17	9	9	20	20	6	16
TeatLength	20	6	5	6	9	8	8	8	17	14	7	13
RumpAngle	21	9	19	14	21	7	7	7	22	11	8	9
UdderDepth	22	17	21	19	12	10	12	11	7	21	10	10

* 1—most important; 22—least important; ME—milking efficiency; AA—average number of teat cup attachments; AT—average time of teat cup attachments; RAI—robotic adaptability index; DIM—days in milk; XGBoost—extreme gradient boosting; RF—random forest; and MLP—multilayer perceptron.

## Data Availability

Data available on request from the authors.

## Abbreviations

The following abbreviations are used in this manuscript:

AMS	Automatic milking system
RAI	Robotic adaptability index
PCA	Principal component analysis
ME	Milking efficiency
